# Minimum Wage and Overweight and Obesity in Adult Women: A Multilevel Analysis of Low and Middle Income Countries

**DOI:** 10.1371/journal.pone.0150736

**Published:** 2016-03-10

**Authors:** Annalijn I. Conklin, Ninez A. Ponce, John Frank, Arijit Nandi, Jody Heymann

**Affiliations:** 1 WORLD Policy Analysis Center, UCLA Fielding School of Public Health, University of California Los Angeles, Los Angeles, California, United States of America; 2 Department of Health Policy and Management, UCLA Fielding School of Public Health, University of California Los Angeles, Los Angeles, California, United States of America; 3 Scottish Collaboration for Public Health Research & Policy, The University of Edinburgh, Edinburgh, Scotland, United Kingdom; 4 Institute for Health and Social Policy and Department of Epidemiology, Biostatistics, and Occupational Health, McGill University, Montreal, Québec, Canada; Hunter College, UNITED STATES

## Abstract

**Objectives:**

To describe the relationship between minimum wage and overweight and obesity across countries at different levels of development.

**Methods:**

A cross-sectional analysis of 27 countries with data on the legislated minimum wage level linked to socio-demographic and anthropometry data of non-pregnant 190,892 adult women (24–49 y) from the Demographic and Health Survey. We used multilevel logistic regression models to condition on country- and individual-level potential confounders, and post-estimation of average marginal effects to calculate the adjusted prevalence difference.

**Results:**

We found the association between minimum wage and overweight/obesity was independent of individual-level SES and confounders, and showed a reversed pattern by country development stage. The adjusted overweight/obesity prevalence difference in low-income countries was an average increase of about 0.1 percentage points (PD 0.075 [0.065, 0.084]), and an average decrease of 0.01 percentage points in middle-income countries (PD -0.014 [-0.019, -0.009]). The adjusted obesity prevalence difference in low-income countries was an average increase of 0.03 percentage points (PD 0.032 [0.021, 0.042]) and an average decrease of 0.03 percentage points in middle-income countries (PD -0.032 [-0.036, -0.027]).

**Conclusion:**

This is among the first studies to examine the potential impact of improved wages on an important precursor of non-communicable diseases globally. Among countries with a modest level of economic development, higher minimum wage was associated with lower levels of obesity.

**Research Highlights**

- Many economic factors are known to influence overweight and obesity, yet evidence on policy-related determinants is scant.
- We examined national minimum wage levels and measured overweight/obesity using multilevel models for 27 developing countries.
- We found a weak but significant lower probability of being overweight or obese associated with higher levels of minimum wage in more developed countries, and a small increase in prevalence in the least developed countries.
- Results suggested a potential societal effect of minimum wage on overweight and obesity that should be examined longitudinally to determine its potential as a structural intervention.

## Introduction

Overweight and obesity pose one of the biggest public health challenges for high, middle and low income countries. The concern for prevention is several-fold. Obesity creates a large disease burden of multiple chronic conditions, affecting the longevity and quality of life of individuals and imposing substantial cost to healthcare systems and wider society;[1] and, levels of obesity have doubled in many countries worldwide in the past two decades, including in low and middle-income countries which have fewer resources to prevent and address the burden.[2]

A key issue is the systematic disparities in obesity across countries and across groups within countries.[2] While high-income countries show a robust negative gradient by individual-level socioeconomic status (SES) and financial hardships in obesity and weight gain,[3–5] the association between SES and BMI or overweight is positive in most developing countries.[3, 5–7] Some of the literature suggests that the social patterning of BMI in developing countries reverses with greater national wealth.[8, 9] In addition, ecological studies show that national economic context (e.g. per capita GDP and globalisation) is also associated with BMI in developing countries, and again the patterns may differ by development stage.[10, 11] Globally, the economic determinants of obesity appear stronger and more consistent in women, and gender differences are exacerbated in developing countries.[12]

For governments to succeed in strategies directed at wider socio-economic determinants of obesity, we must understand whether different types of environments, including policies, are associated with differences in weight status, and whether national-level determinants are independent of individual-level drivers. While there is consensus on economic resources constituting a critical determinant of disparities in overweight and obesity, evidence is lacking on the role of economic-related policies.[13] As one of many ways to ensure a supportive environment, particular interest lies in the role of policies that can be expected to influence a person’s experience of economic (in)security which is associated with obesity.[14] Minimum wage is a policy lever that is associated with body weight and health outcomes in the US context,[15, 16] and could have a profound effect on the lives and health of people living on low wages particularly women who are disproportionately affected by low-wage employment globally.[17] However, its role may be limited for two reasons: first, large proportions of women work in the informal economy typically without minimum wage protection (e.g. 60–80% in sub-Saharan Africa and 30–60% in Latin America);[18] and second, diffusion of cultural ideas and lifestyles has shaped dietary convergence and the desirability of obesogenic fast foods.[19]

By providing a secure income floor, the economic security theory of obesity postulates that minimum wage could affect women’s BMI through lowering physiological stress, which is one of four biological factors linking economic disadvantage to obesity.[20] Multiple simultaneous pathways are possible, and minimum wage could also affect obesity through increasing material resources needed to obtain more calories (in low-income countries) and better quality food (in higher-income countries). While higher minimum wage might help create a more economically secure context in all settings, it could have a different instrumental effect depending on country development and norms. In the poorest countries higher minimum wage could increase women’s ability to consume more calories while in middle-income countries it could increase women’s access to more nutrient-dense foods. However, the impact of minimum wage on food purchasing may be moderated by cultural norms regarding healthy weights that vary across the development spectrum,[12, 21] and by global marketing of fast food that alters perceptions of food quality and prestige value.[19] Using a novel database on nationally legislated minimum wage linked to existing anthropometric data from adult women, we assessed whether minimum wage is indeed related to overweight or obesity prevalence separate from individual-level SES, and whether the direction of association after adjustment for SES differs by country development stage.

## Methods

### Data sources and study sample

We linked national minimum wage data to individual anthropometry and socio-demographics. Data on minimum wage levels came from the minimum wage database developed by McGill University’s Maternal and Child Health Equity (MACHEquity) research program, in collaboration with UCLA’s World Policy Analysis Center. It includes year-specific data on legally mandated minimum wage applying to private sector workers or, if sector-/occupation-specific, to either manufacturing sector or unskilled workers. It was constructed primarily from the ILO Global Wage database for countries with DHS and other international household survey data, and supplemented using additional sources on labor and/or wage legislation. These included: US State Department’s Human Rights Reports; NATLEX and ILO TRAVAIL database of legal documents and memoranda; country-specific government websites; and, in a limited number of cases, reports of business and labor organizations. Other country-level statistics came from the World Bank (World Development Indicators), UNICEF (under-five mortality rates), and Heritage Foundation (Economic Freedom Index) databases. Minimum wage values and country statistics were for the index year when DHS surveys were fielded in a given country.

Individual-level anthropometric and control variables came from the Demographic and Health Survey (DHS) of young and adult women across 34 countries who were interviewed during the period 2004–2006. We chose a recent time period that had more global economic stability than after the financial crisis. The DHS uses a multistage probabilistic sampling process to collect nationally-representative health and wellbeing data (using trained interviewers) for women and their children at regular intervals since 1984 in over a hundred countries, as detailed elsewhere.[22] A total of 462,789 young and adult women (13–49 y) self-reported socio-demographic information and 371,991 were measured objectively for BMI (kg/m^2^). We excluded 163,321 young females (between 13 and 24 years of age), as we were interested in studying adult women of working age with completed education and physiological development. We also excluded 6% of the remaining adult women who reported being pregnant (n = 17,449), leaving 229,066 non-pregnant adult women with anthropometry data.

Of the 34 countries with DHS survey data, there were 27 countries with information on minimum wage (per month, PPP International $) and measured anthropometry in non-pregnant women aged 24–49 years (n = 190,892). We dichotomized BMI (0 = 18.5–24.9 kg/m^2^; 1 = 25 kg/m^2^ and above) to identify women who were overweight (pre-obese) or obese since both are strong risk factors of type 2 diabetes and other serious chronic conditions; we also examined obesity only as an outcome (0 = 18.5–29.9 kg/m^2^; 1 = 30 kg/m^2^ and above). Characteristics of the sample are summarized in S1 Table available online.

### Ethics Statement

A prescribed informed consent statement is read to DHS respondents by the trained interviewer who records whether or not the respondent consented in the questionnaire and then signs to attest that s/he read the consent statement to the respondent. DHS maintains strict standards for protecting privacy and confidentiality of respondents, and procedures were reviewed and approved by the ICF International Institutional Review Board to ensure compliance with the US Department of Health and Human Services regulation for the protection of human subjects (45 CFR 46).[23]

### Statistical methods

Descriptive statistics characterized overweight and obesity, minimum wage and key covariates for all countries and for each country income group. We used pairwise correlation coefficients between minimum wage and country covariates and quantified the variance inflation factor (VIF); minimum wage was strongly correlated with logged GDP (r = 0.715), but no multicollinearity problems were detected (VIF<5 for all independent variables). We calculated the variance partition coefficient to measure the proportion of the total variance due to differences between countries (VPC = level 2 residual variance/level 1 residual variance + level 2 residual variance; where level 1 residual variance is 3.29 for a logit model).

Existing literature informed the selection of economic development factors that could influence levels of minimum wage and distributions of weight. Differences in national (or state) income level are associated with many health outcomes including BMI,[24–26] and countries with higher incomes are likely to set more adequate minimum wage levels. At a given income level, countries also vary widely on public expenditures on health and higher spending is associated with better outcomes.[27] Importantly, less egalitarian (politically polarized) countries invest fewer public resources to create a health infrastructure and so individuals pay large amounts out-of-pocket,[26] whereas more egalitarian countries provide a greater degree of financial protection for the population against major health costs and so the public share of total health expenditures is greater. Countries’ political behaviors towards social protections could also influence policy-making on minimum wage. In addition, the level of regulatory constraint on commerce may separately influence the degree of economic (in)security provided by minimum wage. Importantly, the form of market governance matters for social inequalities in obesity,[28] with more market-liberal regulatory structures being associated with greater mean body weight than a more collectivist approach among high-income countries[24] but potentially less individual overweight in developing counties.[10]

We assessed the cross-sectional association of monthly minimum wage levels (main exposure) with the likelihood of being overweight/obese (main outcome) using multivariable logistic regression with two-level random intercept models (STATA ‘melogit’). Multilevel statistical techniques provide a technically robust framework to account for the hierarchical structure of the data (individuals nested within countries), and are pertinent when predictor variables are measured simultaneously at different levels. We first examined the association of minimum wage with overweight/obesity across all 27 countries and stratified by country income group, with mutual adjustment for all covariates and conventional SES indicators. Thus the remaining odds ratios for overweight/obese (or for obesity only) were interpreted as independent associations of minimum wage. In addition, we used regression coefficients for post-estimation calculation of adjusted prevalence difference for a 1-unit change in minimum wage (STATA ‘margins, dydx($exposure) over(lmic)’). For this, we used the pooled data in multilevel models specified with an interaction term between country minimum wage and income group variables in order to have adequate sample size to provide a meaningful analysis of the average marginal effects of the independent association. Final sample sizes varied (range: 58,930–162,446).

Models controlled for interview year and individual-level socio-demographics known to be associated with weight status and/or economic determinants. These included: age (years); marital status (being currently/previously married; single); parity (having no children; 1–2 children; 3–5 children; 6 or more children (reference)); and tobacco consumption (non-user; user (reference); unknown/missing (e.g. Azerbaijan, Bolivia, Chad, Colombia, Morocco)). Adjustment for individual-level SES included three conventional indicators (education, occupational status and urban location (rural as reference)). Education was defined using four DHS levels, i.e. no education (reference), primary, secondary, and higher. Occupational status was constructed from self-reported occupation group and employment status in the previous 12 months, with categories defined similar to previous research:[29] not working (reference); household, domestic and service workers; agricultural employees and self-employed workers; skilled and unskilled manual workers; and workers in non-manual occupations (professional and managerial; clerical; sales). Models were also conditioned on country-level factors that are associated with our outcome and likely to be related to differences in minimum wage levels: namely, market size (log of per capita GDP, adjusted for PPP in 2011 International $); public sector health spending (as % of total health expenditure); and market-liberal regulatory structure (Economic Freedom Index).

Sensitivity analyses re-estimated the odds ratios to test separate specifications for alternative coding of covariates (i.e. tobacco (y/n), education (years), public sector expenditures on health (as % of GDP); inclusion of other potential confounders (geographic regions; Human Development Index (low/medium/high)); and exclusion of GDP as covariate, or countries with low overweight/obesity prevalence. We also re-examined associations for obesity only. Analyses were performed using STATA v14.0.

## Results

Table 1 shows the distribution of country and individual characteristics for countries overall and by income group. While the average monthly minimum wage (PPP) across all countries was $192 (SD 104), differences were seen between low-income and middle-income countries ($144 (SD 35) versus $295 (SD 126), respectively). The proportions of women across categories of selected characteristics differed substantially between countries by economic development stage, with few exceptions (e.g. mean age, marital status). On average, just over a third of the women in our sample were overweight/obese; proportions were higher in middle-income (56%) than in low-income (26%) countries.

**Table 1:** Descriptive characteristics of study sample of adult women in 27 developing countries.

Mean (SD) or frequency of country and individual characteristics	All countries (n = 27)	Low income countries (n = 17)	Middle income countries (n = 11)
*Country-level*			
Monthly minimum wage, International$	192 (104)	144 (35)	295 (126)
GDP, billions, PPP International $	4120 (2754)	2679 (1101)	7216 (2690)
Log GDP, billions, PPP International $	8.10 (0.70)	7.77 (0.55)	8.81 (0.41)
Under-5 Mortality Rate (U5MR)	70.2 (38.3)	88.0 (31.0)	31.9 (20.0)
Economic Freedom Index (EFI) score	55.3 (4.2)	53.43 (2.8)	59.2 (3.9)
Public sector health spending (% of total health expenditure)	39.2 (19.6)	31.9 (14.4)	54.9 (19.9)
*Individual-level*			
Women, n	162 446	103 516	58 930
Age (24–59 y)	35.0 (7.3)	34.8 (7.2)	35.8 (7.5)
Ever married, n (%)	149 790 (92%)	98 110 (95%)	51 680 (88%)
Parity, n (%)			
0 children	15 609 (10%)	8831 (9%)	6778 (12%)
1–2 children	55481 (34%)	31 891 (31%)	23 590 (40%)
3–5 children	65 243 (40%)	43 069 (42%)	22 174 (38%)
6+ children	26 113 (16%)	19 725 (19%)	6388 (11%)
Tobacco use, n (%)			
Yes	13 311 (8%)	12 006 (12%)	1305 (2%)
No	115 309 (71%)	89 794 (87%)	25 515 (43%)
Unknown/ missing	33 826 (21%)	1716 (2%)	32 110 (54%)
Highest education level, n (%)			
No education	45 794 (28%)	40 991 (40%)	4803 (8%)
Primary	43 888 (27%)	24 875 (24%)	19 013 (32%)
Secondary	55 776 (34%)	29 348 (28%)	26 428 (45%)
Higher	16 988 (10%)	8302 (8%)	8686 (15%)
Occupation group, n (%)			
Not working	65 285 (40%)	45 066 (44%)	20 219 (34%)
Agriculture	34 362 (21%)	29 278 (28%)	5084 (9%)
Services	16 837 (10%)	4950 (5%)	11 887 (20%)
Manual	13 399 (8%)	9360 (9%)	4039 (7%)
Non-manual	32 563 (20%)	14 862 (14%)	17 701 (30%)
Urban location, n (%)	80 727 (50%)	44 617 (43%)	36 110 (61%)
BMI (range: 12.1–59.81 kg/m^2^)	24.4 (4.6)	23.2 (3.9)	26.5 (5.0)
Overweight/ Obese, n (%)	59 398 (37%)	26 404 (26%)	32 994 (56%)
Obese, n (%)	19,211 (12%)	6,797 (7%)	12,414 (21%)

**Fig 1:**
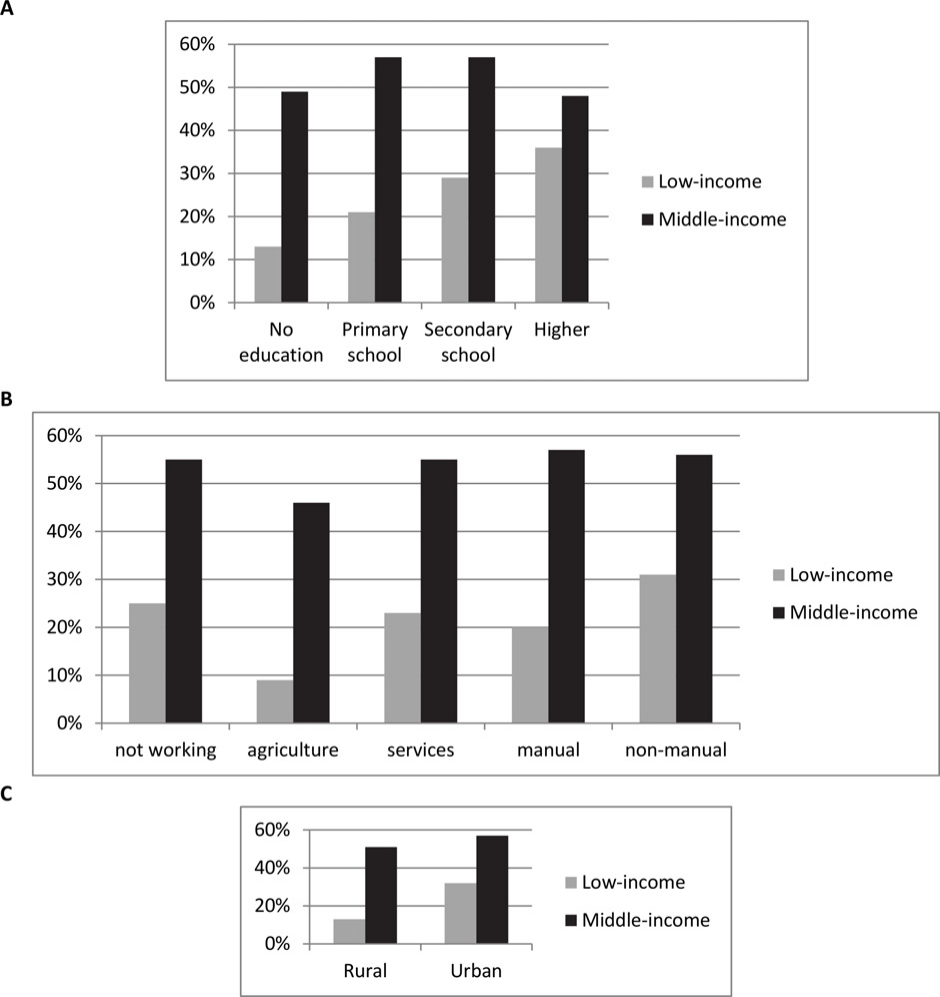
Proportion of overweight and obese women in the sample across categories of socioeconomic status (SES). Panel A. Highest education level. Panel B. Occupation status. Panel C. Geographic location.

More notably, the proportions of women overweight/obese were unevenly distributed across categories of each SES variable (Fig 1). There were large SES differences in women’s overweight prevalence among low-income countries, whereas the social gradient appeared weaker among middle-income countries. SES variation in the outcome specific to each country is also shown in S1 and S2 Figs.

### Independent associations of minimum wage with overweight/obesity and with obesity

In covariate- and SES-adjusted models, we found a significant (p<0.001) but very weak positive association between national minimum wage and overweight/obesity across 27 developing countries (OR 1.0004 [1.0002, 1.0006]) (Table 2). The pattern of independent association, however, differed substantially by country income group. We found a weak positive association (OR 1.0058 [1.0052, 1.0064], p<0.001) in low-income countries, and a weak negative association (OR 0.9996 [0.9993, 0.9998], p = 0.003)) in middle-income countries (Table 2). Sensitivity analyses showed the reversed pattern of stratified results persisted.

**Table 2:** Association of minimum wage with overweight and obesity in adult women in overall and stratified two-level random intercept models.

	All countries		Low-income countries		Middle-income countries	
	OR	(CI95)	OR	(CI95)	OR	(CI95)
Monthly minimum wage, International$	1.0004***	1.0002, 1.0006	1.0058***	1.0052, 1.0064	0.9996**	0.9993, 0.9998
Age (y)	1.06***	1.06, 1.06	1.06***	1.06, 1.06	1.06***	1.06, 1.07
Ever married	1.45***	1.38, 1.54	1.64***	1.49, 1.80	1.38***	1.28, 1.47
0 children	0.95	0.89, 1.01	1.12**	1.03, 1.22	0.91*	0.82, 0.99
1–2 children	1.08***	1.03, 1.12	1.18***	1.12, 1.25	1.14***	1.07, 1.22
3–5 children	1.10***	1.06, 1.14	1.10***	1.05, 1.15	1.34***	1.26, 1.43
Tobacco non-user	1.97***	1.88, 2.06	1.88***	1.78, 1.98	1.20**	1.06, 1.35
Tobacco use unknown/ missing	1.45***	1.36, 1.55	1.75	0.96, 3.18	0.85*	0.74, 0.98
Primary education	1.83***	1.77, 1.89	1.47***	1.40, 1.53	1.39***	1.29, 1.49
Secondary education	1.91***	1.84, 1.97	1.76***	1.69, 1.84	1.29***	1.19, 1.40
Higher education	1.55***	1.48, 1.63	1.89***	1.77, 2.01	0.94	0.86, 1.03
Agriculture occupation	0.46***	0.44, 0.47	0.46***	0.44, 0.48	0.71***	0.66, 0.76
Service occupation	0.78***	0.75, 0.82	0.76***	0.71, 0.82	1.03	0.97, 1.09
Manual occupation	0.85***	0.82, 0.89	0.83***	0.78, 0.87	1.00	0.93, 1.07
Non-manual occupation	1.04*	1.00, 1.07	1.08***	1.03, 1.13	1.18***	1.12, 1.24
Urban	1.51***	1.47, 1.55	2.03***	1.96, 2.10	1.29***	1.23, 1.34
Log of per-capita GDP, International$	1.66***	1.60, 1.72	0.93*	0.86, 0.99	1.33***	1.22, 1.44
Economic Freedom Score	1.03***	1.02, 1.03	0.94***	0.94, 0.95	0.99**	0.98, 0.99
Public spending on health (% of total health expenditures)	1.01***	1.01, 1.01	1.00*	1.00, 1.01	0.99	0.99, 1.00
$\sigma_{u}^{2}$ (Between-country variance)	0.5441	0.1880, 1.5747	0.7757	0.2361, 2.5489	0.0663	0.0038, 1.1582

Odds ratios (95% CI) obtained by two-level random intercept model. Sample restricted to adult women (24–49 y). Number of observations were: All, n = 162,446; Low, n = 103,516; Middle, n = 58,930. Reference groups for each set of control variables were: women having 6 or more children, tobacco users, no education, being unemployed, and living in a rural location.

*** p<0.001

** p<0.01

* p<0.05.

By contrast, the association of country GDP (logged) with the outcome was much stronger and was negative in low-income countries but positive in middle-income countries (Table 2). And, as expected, there was a monotonic increase in the association between education and the outcome in low-income countries; while in middle-income countries, the opposite was observed. The residual variation in the odds of being overweight/obese that is attributable to unobserved country characteristics was 14% for the overall model (VPC = 0.142); 19% for the low-income stratified model (VPC = 0.19) and 2% for the middle-income stratified model (VPC = 0.019).

Results were different for obesity as the overall association showed a negative direction; was non-significant in low-income countries; but remained negative and stronger in middle-income countries with higher minimum wage associated with significantly less obesity (S2 Table). Logged GDP was no longer significant in stratified models but higher education was still associated with higher obesity in low-income countries and with lower obesity in middle-income countries. Notably, we found higher percentages of residual variance in the odds of obesity attributable to unobserved country differences than we did for overweight/obesity.

**Fig 2:**
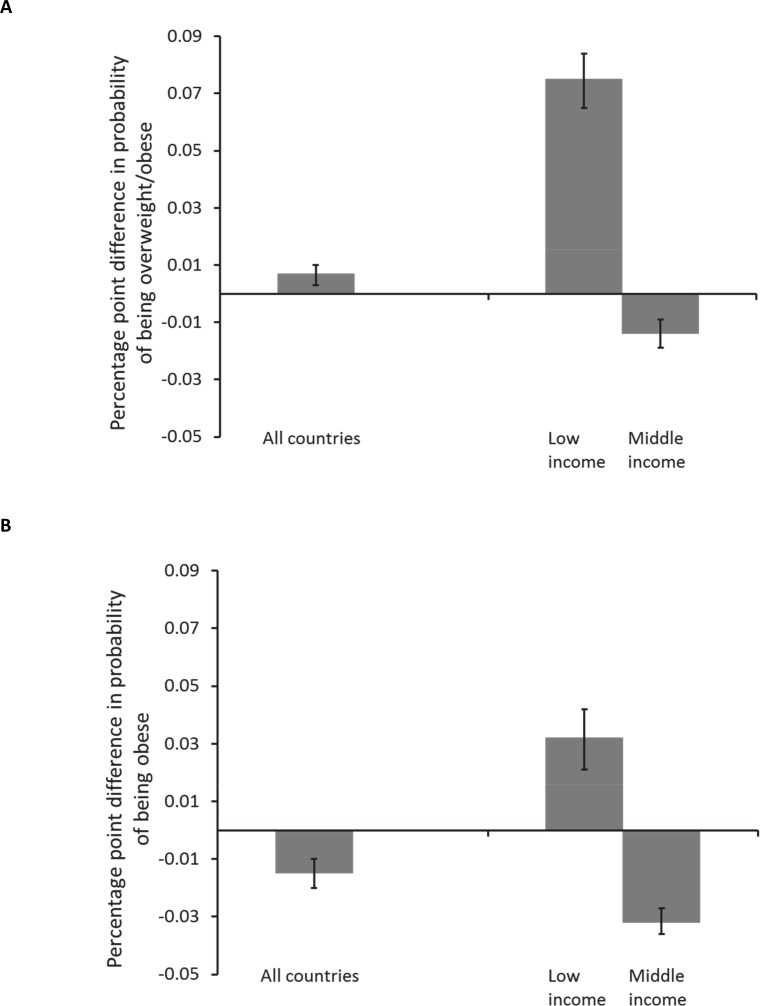
**Adjusted differences in probability of being overweight/obese (panel A) or being obese (panel B) associated with a 1% increase in monthly minimum wage using pooled data.** Post-estimation calculation of average marginal effects using pooled data in multivariable multilevel models with interaction term between minimum wage and country income group (n = 162 446).

In post-estimation using the full sample, we found a 1% increase in minimum wage was associated with an average increase of about 0.1 and 0.03 percentage points in the predicted probability of overweight/obesity and obesity, respectively, in low-income countries (Fig 2). In middle-income countries, we calculated an average decrease of approximately 0.01 and 0.03 percentage points in the predicted probability of overweight/obesity and obesity for a 1% increase minimum wage (Fig 2). Detailed estimates are given in S3 and S4 Tables.

## Discussion

This cross-sectional study of 27 developing countries used a novel dataset to find very small associations between national minimum wage and individual women’s overweight and obesity, after controlling for known confounders including individual-level SES. In particular, results showed a reversed pattern of association by country development stage, with higher levels of minimum wage appearing protective against overweight and obesity among adult women living in countries with middle income. Findings are suggestive of a potential social-level effect of minimum wage, although the relative contribution was minimal compared to individual-level SES and country income level.

Evidence is generally sparse regarding the role of policy as a social determinant of health,[13] and employment conditions affecting weight.[30] There are no studies to our knowledge of minimum wage in relation to overweight and obesity among adult women in a developing context. Our results for middle income countries echo the US studies. Existing research on US adults using survey data over a timeframe including our study period (1984–2006) demonstrates an inverse association between inflation-adjusted minimum wage and self-reported mean BMI and that associations are stronger at the higher end of the distribution.[16] Meltzer and Chen (2011) further show how declines in real minimum wage of US$0.68 (end of their study period) and US$3.33 (before their study period) explain 4% and 10% of the total increase in average BMI. Another study using the same survey data (1996–2007) shows potential protective effects of higher state-level minimum wage for self-reported health status.[15] Furthermore, our finding of a reversed pattern of association with overweight and obesity by country development stage parallels other work using DHS data. Another multilevel international study reported protective effects of low individual SES on women’s obesity in low-income countries and insalubrious effects in upper-middle income countries.[8] And, in a recent econometric study using single-level fixed effects models over a longer period (1991–2009), the relationship between economic globalization and overweight in young and adult women also changed direction from positive in the least economically globalized countries to negative in the most economically globalized.[10]

Our finding of small magnitudes of association could have resulted from a combination of a large relationship among women in low-wage occupations with minimum wage protection and no relationship (as would be expected) among women unaffected by the minimum wage either because they were in the informal economy (typically without a minimum fixed wage) or because their salaries were already above the legislated level. There are also multiple potential reasons for the mixed pattern in the direction of association by country development stage. It is possible that in least developed countries, minimum wage is relevant only to a small group of women who are employed in the formal economy (or in the informal sector)[18] and use their secure income to buy energy-dense calories, or who belong to elite social groups that are known to have higher weight status as a result of patterns of low-energy expenditure, lack of food insecurity and cultural values of wealth/prestige favoring large body shape.[5, 12] Given we adjusted for individual SES, results for the least developed countries might be explained by other broader factors such as technological change, infrastructure availability, wars, famine and economic/climate shocks could each affect both minimum wage and overweight levels.[10, 31]

By contrast, national minimum wage may apply to a wider segment of employed women in the population after a certain stage of development expressed by country per capita GDP. However, several of the middle-income countries examined also have high proportions of women employed in the informal economy.[32] Nevertheless, the informal economy in relatively more developed countries may have closer or stronger linkages to formal regulatory environment such as minimum wage protection due to different types of production systems,[18] and to greater awareness among workers of their rights to certain legal and social protection.[32] Thus, after considering women’s SES, higher levels of national minimum wage may protect women against overweight and obesity through effects on food security which is consistently associated with obesity disparities in women,[33] and/or on perceived economic security which has stress-related effects on BMI.[14, 28] It bears noting that average calorie intake, exercise, smoking and alcohol are important, but do not fully explain the social gradients in obesity in women in developed countries;[34, 35] hence stress-related factors are increasingly proposed as plausible mediators.[4, 36]

Finally, the role of minimum wage in disparities of overweight and obesity may be modified (rather than confounded) by individual SES at any country development level,[9, 11] and such potential interactions should be further investigated. It is worth noting that we found a strong positive relationship between education and obesity outcomes in low-income countries and a strong negative relationship in middle-income countries, which is consistent with the wider literature.[3, 12] Separately, there was also a differing pattern of association between different occupation categories and obesity outcomes by country development stage, particularly regarding the role of service and manual occupations which were strongly protective in low-income, but not middle-income, countries. The latter might suggest that service and manual occupations might differ in the composition of job types and/or in the nature of work as countries develop. Nevertheless, across development stages, agricultural work was unsurprisingly negatively associated with overweight/obesity while non-manual occupations were positively associated with overweight/obesity.

### Limitations

Study limitations include the cross-sectional nature of the data, which limits causal inference and does not unpack the influence of secular trends in obesity. Absence of reliable data on informal sector workers covering the countries studied, and lack of information on policy enforcement and implementation reach are further limitations. We also cannot investigate gender differences or differences between mandated minimum and prevailing market wage. Moreover, there is potential for residual confounding from individual income not measured in the DHS, and from above-mentioned unobserved country factors. However, we have addressed some important confounding by including three economic development factors that may explain some unmeasured labor market features. Notably, multilevel models also help to address some unobserved natural heterogeneity across countries. Approximately 20% of adult women in the sample were dropped in analyses due to missing anthropometry data which may have induced non-response/ selection bias. And, the small sample of middle-income countries in stratified analysis means that estimates are likely based on extrapolation and SES might be underestimated.

### Strengths

Several strengths of our study favor the validity of our findings: the nationwide probabilistic samples and large number of observations; comparability of anthropometric outcomes; range of country economic development level and geographical regions; highly standardized data collection procedures; multiple potential confounders; and, appropriate multilevel analyses. Stratified results were robust to alternative model specifications, and the associations between individual-level SES and the outcome also differed by country development stage and revealed a pattern consistent with the literature.

## Conclusion

A clear link exists between national minimum wage and overweight measured in adult women in developing countries. The societal phenomenon observed showed a reversed pattern by country development stage. Future work needs to use longitudinal analyses to show the potential impact of changing minimum wage so as to determine whether this employment policy might serve as a possible structural intervention for the growing burden of excess weight among women across all social groups.

## Supporting Information

S1 Fig Differences by SES in the proportion of overweight and obese women in each low-income country. Panel A. Highest education level. Panel B. Occupation status. Panel C. Geographic location.
(PDF)

S2 Fig Differences by SES in the proportion of overweight and obese women in each middle-income country. Panel A. Highest education level. Panel B. Occupation status. Panel C. Geographic location.
(PDF)

S1 Table Adverse anthropometric outcomes, monthly minimum wage and level of development in the study sample of adult women, by country. (PDF)

S2 Table Association of minimum wage with obesity in adult women in overall and stratified two-level random intercept models. (PDF)

S3 Table Association of monthly minimum wage with overweight and obesity in adult women using pooled data with interaction terms. (PDF)

S4 Table Association of monthly minimum wage with obesity in adult women using pooled data with interaction terms. (PDF)
