# Supplementary material for: Minimum Wage and Overweight and Obesity in Adult Women: A Multilevel Analysis of Low and Middle Income Countries
Source: PLoS One. 2016 Mar 10;11(3):e0150736. doi: 10.1371/journal.pone.0150736 (PMC4786275; doi:10.1371/journal.pone.0150736)
Supplement: S2 Fig — Panel A. Highest education level. Panel B. Occupation status. Panel C. Geographic location. (PDF) [file pone.0150736.s002.pdf]

A

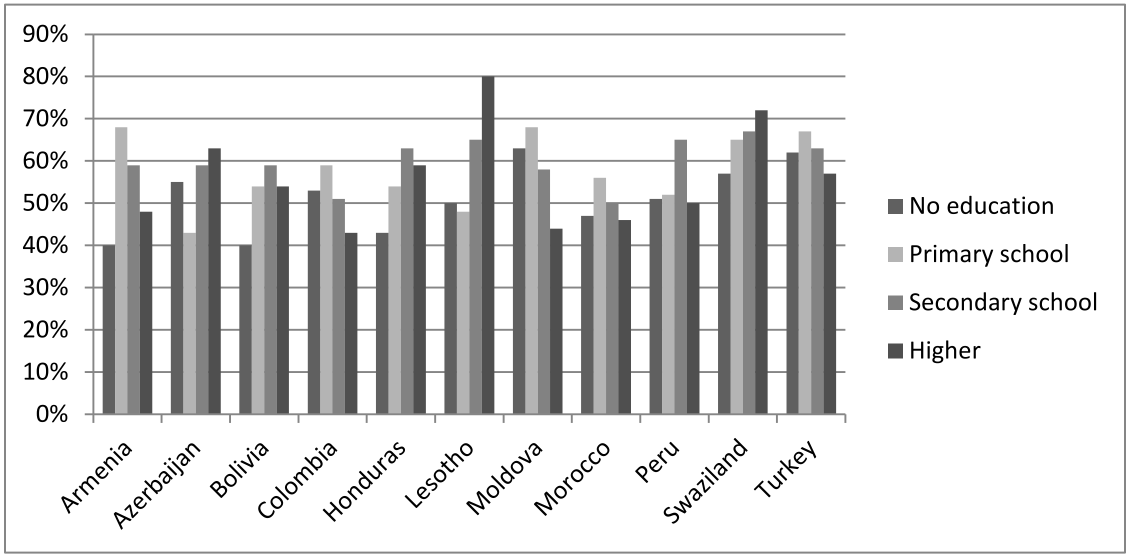

90%
80%
70%
60%
50%
40%
30%
20%
10%
0%
Armenia
Azerbaijan
Bolivia
Colombia
Honduras
Lesotho
Moldova
Morocco
Peru
Swaziland
Turkey
No education
Primary school
Secondary school
Higher

B

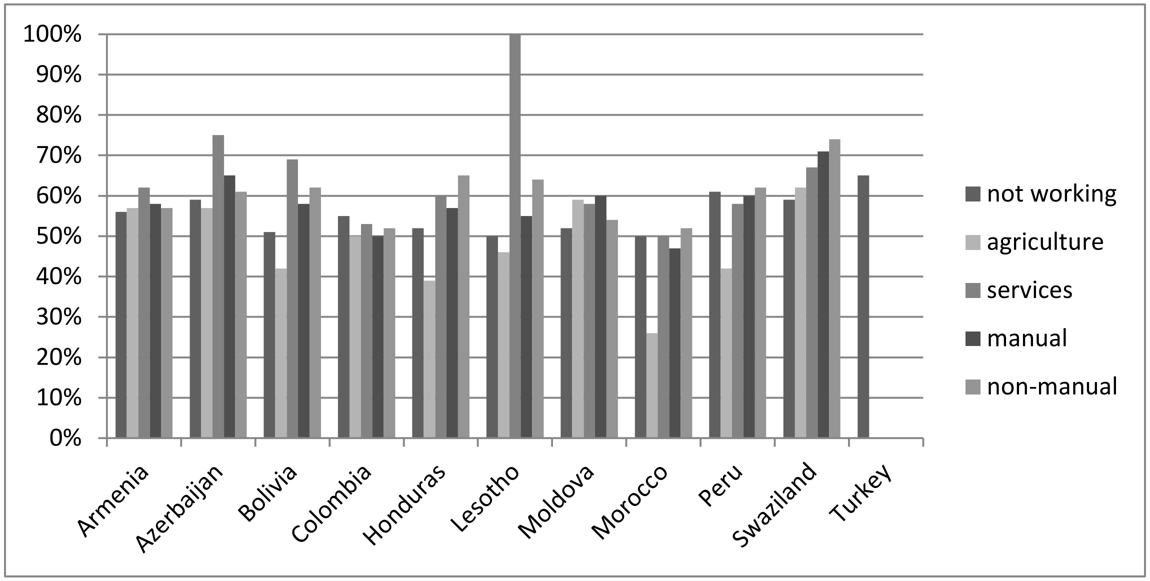

100%
90%
80%
70%
60%
50%
40%
30%
20%
10%
0%
Armenia
Azerbaijan
Bolivia
Colombia
Honduras
Lesotho
Moldova
Morocco
Peru
Swaziland
Turkey
not working
agriculture
services
manual
non-manual

C

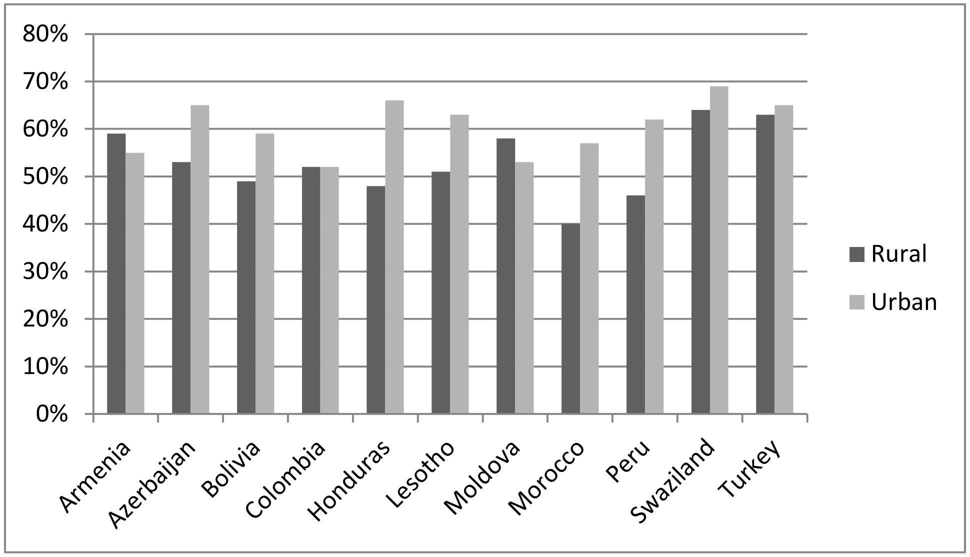

S2Fig Differences by SES in the proportion of overweight and obese women in each middle-income country

Note: very high proportions in some country categories resulted from very small numbers of women
