# Supplementary material for: Minimum Wage and Overweight and Obesity in Adult Women: A Multilevel Analysis of Low and Middle Income Countries
Source: PLoS One. 2016 Mar 10;11(3):e0150736. doi: 10.1371/journal.pone.0150736 (PMC4786275; doi:10.1371/journal.pone.0150736)
Supplement: S1 Table — (PDF) [file pone.0150736.s003.pdf]

S1 Adverse anthropometric outcomes, monthly minimum wage and level of development in the study sample of adult women, by country

	N	Year of interview	Mean (SD) BMI	Underweight, N (%)	Overweight or Obese, N (%)	Minimum Wage	Income group	EFI score	High U5MR
Armenia	4305	2005	26.6 (5.5)	104 (2%)	2426 (56%)	82.51	Low-Middle	69.8	No
Azerbaijan	5514	2006	26.7 (5.1)	122 (2%)	3274 (59%)	133.63	Low-Middle	53.2	No
Bolivia	1250	2004	26.3 (4.8)	14 (1%)	667 (53%)	223.87	Low-Middle	64.5	No
Cambodia	3352	2005	21.3 (3.2)	582 (17%)	440 (13%)	166.58	Low	60.0	No
Cambodia	1742	2006	21.3 (3.4)	330 (19%)	219 (13%)	182.81	Low	56.7	No
Cameroon	2696	2004	24.4 (4.6)	131 (5%)	948 (35%)	104.59	Low	52.3	Yes
Chad	2071	2004	21.4 (3.6)	406 (20%)	261 (13%)	146.55	Low	52.1	Yes
Colombia	5631	2004	26.0 (4.7)	134 (2%)	3070 (55%)	384.75	Low-Middle	61.2	No
Colombia	17 688	2005	25.8 (4.8)	500 (3%)	9134 (52%)	400.88	Low-Middle	59.6	No
Ethiopia	3706	2005	20.6 (3.3)	930 (25%)	292 (8%)	93.37	Low	51.1	Yes
Haiti	1151	2005	22.8 (4.7)	175 (15%)	275 (24%)	139.69	Low	48.4	No
Haiti	1745	2006	23.5 (4.6)	172 (10%)	527 (30%)	125.47	Low	49.2	No
Honduras	2581	2005	26.7 (5.2)	46 (2%)	1473 (57%)	231.43	Low-Middle	55.3	No
Honduras	8695	2006	26.4 (5.3)	181 (2%)	4736 (54%)	251.32	Low-Middle	57.4	No
India	4570	2005	21.5 (4.5)	1285 (28%)	918 (20%)	155.34	Low	54.2	No
India	70 766	2006	21.8 (4.4)	17 382 (25%)	14 695 (21%)	150.45	Low	52.2	No
Lesotho	1755	2004	26.3 (5.9)	64 (4%)	911 (52%)	240.08	Low	50.3	Yes
Lesotho	138	2005	26.1 (5.4)	6 (4%)	76 (55%)	241.40	Low-Middle	53.9	Yes
Liberia	284	2006	23.4 (4.4)	19 (7%)	77 (27%)	138.14	Low	48.1†	Yes
Madagascar	3476	2004	21.7 (3.4)	495 (14%)	471 (14%)	131.43	Low	60.9	No
Malawi	4456	2004	22.3 (3.3)	351 (8%)	718 (16%)	39.65	Low	53.6	Yes
Malawi	1486	2005	22.6 (3.8)	122 (8%)	283 (19%)	63.87	Low	53.6	Yes
Mali	7934	2006	22.9 (4.5)	791 (10%)	1905 (24%)	157.06	Low	54.1	Yes
Moldova	4825	2005	26.8 (5.8)	109 (2%)	2658 (55%)	154.80	Low-Middle	57.4	No
Morocco	2928	2004	25.5 (4.5)	101 (3%)	1423 (49%)	501.26	Low-Middle	56.7	No
Mozambique	65	2004	20.9 (2.2)	7 (11%)	2 (3%)	103.82	Low	57.2	Yes
Nepal	6412	2006	20.9 (3.3)	1482 (23%)	698 (11%)	206.93	Low	53.7	No
Niger	2554	2006	22.7 (4.4)	345 (14%)	610 (24%)	105.98	Low	52.5	Yes
Peru	3805	2005	26.1 (4.3)	31 (1%)	2113 (56%)	348.79	Low-Middle	61.3	No
Senegal	2396	2005	23.3 (4.9)	278 (12%)	695 (29%)	166.95	Low	57.9	No
Swaziland	2014	2006	28.3 (6.2)	23 (1%)	1323 (66%)	137.43	Low-Middle	61.4	Yes

Tanzania	3924	2004	22.9 (4.3)	368 (9%)	888 (23%)	133.74	Low	60.1	No
Tanzania	1802	2005	22.5 (4.1)	206 (11%)	370 (21%)	129.70	Low	56.3	No
Turkey	1613	2004	27.4 (5.2)	17 (1%)	1037 (64%)	546.64	Upper-Middle	52.8	No
Uganda	1562	2006	22.3 (4.0)	194 (12%)	288 (18%)	9.95	Low	63.9	Yes

PPP-adjusted minimum wage level per month and country statistics reported for the year index women were interviewed in a given country († value first available for 2009). EFI, Economic Freedom Index. U5MR, Under-5 Mortality Rate (high=100+ deaths/1,000 live births).
