# Supplementary material for: Minimum Wage and Overweight and Obesity in Adult Women: A Multilevel Analysis of Low and Middle Income Countries
Source: PLoS One. 2016 Mar 10;11(3):e0150736. doi: 10.1371/journal.pone.0150736 (PMC4786275; doi:10.1371/journal.pone.0150736)
Supplement: S2 Table — (PDF) [file pone.0150736.s004.pdf]

S2 Table Association of minimum wage with obesity in adult women in overall and stratified two-level random intercept models

	All countries		Low-income countries		Middle-income countries	
	OR	(CI95)	OR	(CI95)	OR	(CI95)
Monthly minimum wage, International$	0.9985***	0.9981, 0.9989	0.9979	0.9928, 1.003	0.9983***	0.9980, 0.9987
Age (y)	1.06***	1.06, 1.07	1.07***	1.07, 1.08	1.06***	1.06, 1.06
Ever married	1.40***	1.29, 1.52	1.72***	1.45, 2.03	1.35***	1.22, 1.48
0 children	0.90*	0.82, 0.98	1.32***	1.14, 1.52	0.87*	0.77, 0.98
1—2 children	0.88***	0.83, 0.93	1.15**	1.05, 1.26	0.95	0.88, 1.03
3—5 children	1.04	0.99, 1.09	1.21***	1.11, 1.31	1.21***	1.13, 1.30
Tobacco non-user	1.95***	1.80, 2.12	1.98***	1.77, 2.21	1.07	0.94, 1.23
Tobacco use unknown/ missing	1.49***	1.33, 1.67	1.51	0.52, 4.35	0.87	0.73, 1.04
Primary education	2.02***	1.91, 2.13	1.51***	1.39, 1.64	1.17***	1.07, 1.28
Secondary education	2.06***	1.95, 2.18	2.03***	1.88, 2.20	1.13*	1.03, 1.25
Higher education	1.58***	1.47, 1.70	2.01***	1.81, 2.24	0.85**	0.76, 0.96
Agriculture occupation	0.39***	0.37, 0.42	0.32***	0.28, 0.35	0.68***	0.62, 0.74
Service occupation	0.85***	0.80, 0.90	0.78***	0.69, 0.89	1.03	0.97, 1.11
Manual occupation	0.90***	0.84, 0.95	0.78***	0.71, 0.85	0.95	0.88, 1.04
Non-manual occupation	1.04	0.99, 1.09	0.93	0.87, 1.00	1.15***	1.09, 1.23
Urban	1.52***	1.46, 1.57	2.45***	2.30, 2.61	1.40***	1.34, 1.48
Log of per-capita GDP, International$	1.35***	1.26, 1.45	0.55	0.21, 1.46	0.94	0.83, 1.07
Economic Freedom Score	1.02***	1.02, 1.03	0.91***	0.87, 0.94	0.98***	0.97, 0.99
Public spending on health (% of total health expenditures)	1.01***	1.01, 1.02	1.02	0.99, 1.06	1.01***	1.00, 1.01
σ_u^2 (Between-country variance)	0.8453	0.4215, 1.6952	2.1437	0.9654, 4.7602	0.1980	0.0339, 1.1580
Variance partition coefficient	0.204		0.395		0.057	

Odds ratios (95% CI) obtained by two-level random intercept model. Sample restricted to adult women (24-49 y). Number of observations were: All, n=162,446; Low, n=103,516; Middle, n=58,930. Reference groups for each set of control variables were: women having 6 or more children, tobacco users, no education, being unemployed, and living in a rural location. ***p<0.001, **p<0.01, *p<0.05
