# Supplementary material for: Minimum Wage and Overweight and Obesity in Adult Women: A Multilevel Analysis of Low and Middle Income Countries
Source: PLoS One. 2016 Mar 10;11(3):e0150736. doi: 10.1371/journal.pone.0150736 (PMC4786275; doi:10.1371/journal.pone.0150736)
Supplement: S3 Table — (PDF) [file pone.0150736.s005.pdf]

S3 Table Association of monthly minimum wage with overweight and obesity in adult women using pooled data with interaction terms

	All countries		Low-income countries		Middle-income countries	
Odds ratios (CI95)	1.0004***	1.0002, 1.0006	1.0045***	1.0040, 1.0050	0.9993***	0.9991, 0.9996
Predicted prevalence difference	.00007***	.00003, .0001	.00075***	.00065, .00084	-.00014	-.00019, -.00009

Odds ratios (95% CI) obtained by two-level random intercept model. Development-specific estimates with interaction term between minimum wage (continuous) and income group (binary). Sample restricted to adult women (24-49 y), n =162 446. Level 2 residual variance was =.3102 (SE .1743). Variance Partition Coefficient (VPC) = 0.086. Post-estimation of predicted probability of being overweight with 1-unit change in minimum wage obtained using average marginal effects with respect to minimum wage over country income group. ***p<0.001, **p<0.01, *p<0.05.
