# Supplementary material for: Minimum Wage and Overweight and Obesity in Adult Women: A Multilevel Analysis of Low and Middle Income Countries
Source: PLoS One. 2016 Mar 10;11(3):e0150736. doi: 10.1371/journal.pone.0150736 (PMC4786275; doi:10.1371/journal.pone.0150736)
Supplement: S4 Table — (PDF) [file pone.0150736.s006.pdf]

S4 Table Association of monthly minimum wage with obesity in adult women using pooled data with interaction terms

	All countries		Low-income countries		Middle-income countries	
Odds ratios (CI95)	0.9985***	0.9981, 0.9989	1.0043***	1.0029, 1.0057	0.9979***	0.9976, 0.9982
Predicted prevalence difference	-.00015***	-.0002, -.0001	.00032***	.00021, .00042	-.00032	-.00036, -.00027

Odds ratios (95% CI) obtained by two-level random intercept model. Development-specific estimates with interaction term between minimum wage (continuous) and income group (binary). Sample restricted to adult women (24-49 y), n =162,446. Level 2 residual variance was 0.6538 (SE .233). Variance Partition Coefficient (VPC) = 0.166. Post-estimation of predicted probability of being overweight with 1-unit change in minimum wage obtained using average marginal effects with respect to minimum wage over country income group. ***p<0.001, **p<0.01, *p<0.05.
